# The association between under-nutrition, school performance and perceptual motor functioning in first-grade South African learners: The North-West Child Health Integrated with Learning and Development study

**DOI:** 10.4102/hsag.v24i0.1046

**Published:** 2019-09-25

**Authors:** Anita E. Pienaar

**Affiliations:** 1 Focus area of PHaSRec, Faculty of Health Science, North-West University, Potchefstroom, South Africa

**Keywords:** underweight, wasting, stunting, motor functioning, academic performance

## Abstract

**Background:**

Early childhood is characterised by an immense spurt of growing and learning where under-nutrition can have adverse effects on the neuro-developmental health and school performance of children. A full understanding of the relationship between school performance and motor functioning skills and malnourishment in school beginners is still lacking.

**Aim:**

To determine the association between indices of under-nutrition and how it relates to school performance and motor functioning skills of first-grade learners.

**Setting:**

North West province (NWP) of South Africa (SA).

**Method:**

The baseline data of the stratified, randomised North-West Child Health Integrated with Learning and Development (NW-CHILD) longitudinal study were used. Grade 1 learners (*N* = 816, 420 boys, 396 girls, mean age 6.78+ years) from four school districts in the NWP of SA took part in the study. Indices of under-nutrition were determined by *Z*-scores (−2 standard deviation [s.d.]) for stunting (height-for-age [HAZ]) and wasting and underweight (*Z*-score for body mass index) using the 2007 World Health Organization reference sample. The Bruininks–Oseretsky Test of Motor Proficiency Short Form and the Visual Motor Integration fourth edition were used to assess different aspects of motor functioning, while school performance in mathematics, reading and writing was assessed by teachers according to the National South African standards of assessments.

**Results:**

Both HAZ and *Z*-score for weight-for-age correlated significantly with school performance and motor functioning skills (*r* > 2.0, *p* < 0.05), while visual perception was moderately associated (*r* < 0.30) with mathematics in HAZ and *Z*-score for weight-for-height (WHZ) children. Motor functioning of HAZ and WHZ children was significantly poorer (*p* < 0.05) compared to typical children, while underweight was not associated with any outcome variables.

**Conclusion:**

Moderate forms of stunting and wasting influence school performance and motor functioning of school beginners negatively, while an association between visual perceptual abilities and inferior mathematics, reading and writing suggests a close link with inferior cognitive information processing in stunted and wasted children. These barriers should be addressed as poor scholastic success in Grade 1 may influence future school performance and the subsequent well-being of children.

## Introduction

Many children younger than 5 years old in developing countries are exposed to multiple risks, including malnutrition, poverty, poor health and poorly stimulating home environments that could detrimentally affect their cognitive, motor and socio-emotional development. This could lead to adverse neuro-developmental and health outcomes (Grantham-McGregor et al. 2007; Sudfeld et al. 2015). Adequate nutrition is one of the most important determinants of children’s health, growth and development and the main cause of under-nutrition, as a healthy diet supports normal growth and development in children (Nyaradi et al. 2013). Under-nutrition is categorised as stunting which is a deficit in height-for-age (HAZ), wasting which is a deficit weight-for-height (WHZ) and underweight which is a deficit in weight-for-age (WAZ) (World Health Organization [WHO] 2007). Although these conditions are described separately, they frequently co-exist and lead to compounded risk (Prendergast & Humprey 2014).

Under-nutrition contributes to various health and developmental risks among children, including extreme loss of body mass, stunted growth and poor resistance against infections. Linear growth failure is the most prevalent form of under-nutrition globally (Prendergast & Humprey 2014), which is mostly attributed to the high sensitivity of increases in height to food security and nutrition deficiencies (Dewey & Begum 2011). Stunting is also one of the major risk factors together with inadequate cognitive stimulation, iodine and iron anaemia for failure to attain full developmental potential (Nyaradi et al. 2013). A child’s brain development is closely related to nutritional status, especially during the early childhood years. Benton (2010) reported that nutrient deficiencies in children in developing countries during critical stages of cognitive development have long-term effects on their intellectual development. These critical stages refer to a phase where certain environmental stimuli facilitate cognitive development and where the body is vulnerable towards these stimuli. These risk factors can become chronic and contribute to impaired cognitive functioning over the longer term because of damage to the brain, and premature death in worst cases (Dewey & Begum 2011). Under-nutrition shows relationships with school performance and is also related to mental capacity (Grantham-McGregor et al. 2007; Milman et al. 2005). Milman et al. (2005) reported impaired intelligence, psychomotor development, fine motor skills and poor neuro-sensory integration as a result of stunting during childhood which are all related to mental capacity and academic achievement, while underperformance in tests of attention, working memory, learning and memory is also documented (Kar, Rao & Chandramouli 2008). Undernourished children were also not school-ready when they entered Grade 1 and had to repeat this school grade as reported by Benton (2010). According to this researcher, brain growth is faster than the rest of the body during the early years (Benton 2010), which may make it more vulnerable to dietary deficiencies during this period.

Early childhood is a period of incredible growth and learning. The development of motor control and coordination is an important part of general development where the first few years of life are particularly important for gross motor development. This developmental period coincides with the time period of significant brain growth (Nyaradi et al. 2013) as the optimal developmental period for basic motor skills spans from before birth to the age of 5 years. However, the importance of motor development goes beyond the attainment of new motor skills as there is a growing body of evidence that confirms a mutual association between cognition and motor skills (Abe & Hanakawa 2009; Hanakawa 2011; Weaver 2003). Neuro-imaging studies have shown that regions important to motor functioning and cognition such as the cerebellum, dorsolateral prefrontal cortex and the connecting structures, including the basal ganglia, are co-activated in certain motor and cognitive tasks, which confirms the mutual association between these two domains (Abe & Hanakawa 2009; Hanakawa 2011), suggesting that these neuro-anatomical areas are interconnected. Furthermore, areas of the brain implicated in language functions are also activated during motor tasks. Van der Fels et al. (2015) reported that although motor, cognitive and language development may share several underlying processes, fine motor skills show the strongest relationship with higher order cognitive skills in their study. Cheung, Yip and Karlberg (2001) reported that thinness during birth and postnatal stunting showed linear inverse relationships with gross motor development of children, while these researchers also confirmed that under-nutrition affects areas of brain development involved in cognition, memory and locomotor skills. Benefice et al. (1996) also confirmed an association between under-nutrition and motor development and indicated that the effect may vary according to the severity, duration and the timing of nutritional deprivation.

Visual perception (VP) is considered to be part of a complex set of mental functions needed for cognition (Bezrukikh & Terebova 2009; Nyaradi et al. 2013), and the period between 5 and 7 years is highlighted as a sensitive developmental period during the maturation of different VP mechanisms. Poorer performance on memory, visual processing and visio-spatial tasks is reported (Davis, Pitchford & Limback 2011; Laus et al. 2011), while Milman et al. (2005) confirmed relationships with psychomotor development, handwriting speed and neuro-sensory integration in children with growth deficits. Studies have also shown that the maturation of specific brain areas during childhood is associated with development of specific cognitive functions such as language, reading and memory (Giedd et al. 2010; Nagy, Westerberg & Klingberg 2004).

Under-nutrition is a worldwide epidemic affecting 156 million children under the age of 5 years who are stunted, 67% of whom are suffering from the most severe forms of acute under-nutrition (50% wasted, 17% severely wasted), while 42 million children are underweight. In low to middle sub-Saharan African countries, statistics reveals that 21.2% of children below 5 years are stunted, 8.2% moderately wasted, 2.6% severely wasted and 12.2 % are underweight (Global Nutrition Report 2016). Statistics further indicate that under-nutrition and stunting are also very prevalent in South African children where 23.9% of all children under the age of 5 years are stunted, 8.7% are underweight and 4.7% have deficit WHZ. Improvement of this epidemic still seems to be marginal (UNICEF, WHO & World Bank 2016), and countries in Africa show the smallest decrease (23% – 17%).

The above background provides evidence of relationships between under-nutrition, school performance and motor functioning of young children which pose a threat for their neuro-developmental cognition and health, not only in the short term, but also for their long-term health and well-being, and these once again also have consequences for society as a whole. South Africa (SA) is a low- to middle-income country (LMIC) with high percentages of the child population who grow up in low socio-economic environments which place them at high risk of the detrimental effects of under-nutrition. National statistics provided by the SANHANES-1 study (Shisana et al. 2014) indicate that stunting is the largest form of under-nutrition in SA and the North West province is highlighted as one of the nine provinces in SA characterised by high percentages of under-nutrition (Shisana et al. 2014).

The early years of life are critical for physical growth and broader cognitive, motor and socio-emotional development but the link between these processes still remains unclear. More understanding is also still needed about how motor and visual perceptual functioning skills may relate not only to academic difficulties of children with under-nutrition, but also the effects of under-nutrition on children’s neuro-development as seen in their school performance to fully understand the magnitude of these influences on young children’s overall development. Such understanding can guide different stakeholders to develop preventative strategies with regard to the negative influences of nutritional impairments on the overall development of young children.

To date similar studies had mostly been focussed on the detrimental effects of under-nutrition during the first 5 years of life. A few studies (Gandhi et al. 2011; Glewwe, Jacoby & King 2001; Haile et al. 2016; Themane et al. 2003) have investigated the relationships between under-nutrition and academic performance in school-age children, although no studies could be found that considered the influences of motor and visual perception functioning skills in South African school beginners while also investigating the possible link between motor and perceptual functioning and school performance of under-nourished children. The aims of this study were therefore twofold, viz. firstly to determine whether under-nutrition is associated with the school performance of under-nourished school beginners and secondly to investigate the possible interplay between under-nutrition and different motor and perceptual functioning skills and the school performance of Grade 1 learners in the North West province of SA.

## Materials and methods

### Research design

This study utilised a quantitative research method and made use of cross-sectional data within a longitudinal research design.

### Research group

The research formed part of the North-West Child Health Integrated with Learning and Development (NW-CHILD) study. This is a 7-year longitudinal study that includes baseline measurements and two-point follow-up measurements which were 3 years apart (2010, 2013 and 2016). The baseline measurements that included Grade 1 learners in the North West province of SA served as the target population for this study. The total number of participants selected for inclusion was 880 Grade 1 learners. The participants were selected randomly by means of stratification by school district and gender. To determine the research group, a list of names of public schools in the North West province was obtained from the Department of Basic Education. From the list of schools in the North West province, which are grouped in eight education districts, each representing 12–22 regions, with approximately 20 schools (minimum 12, maximum 47) per region, school districts and schools were selected randomly with regard to population density and school status – quintile (Q) 1, that is, schools from very poor economic sectors to Q5, that is, schools from very good economic sectors. Twenty schools, from four school districts with a minimum of 40 children per school and with an even gender distribution, were involved in the study. The total group that participated consisted of 816 learners (419 boys and 397 girls) with a mean age of 6.78 years (6.0–7.67 years) and an ethnic distribution of 567 black learners (69.5%), 218 white learners (26.7%), 20 mixed-race learners (2.5%) and 11 Indian (1.3%) learners. Sixty-four of the selected children were not tested. The parents of 13 (1.5%) of the children did not consent for them to participate, while the rest were absent from school on the day of testing or had to be excluded because of incorrect ages being provided by the schools. The number and gender of children in the different quintile schools were Q1 (*N* = 155; 79 boys, 76 girls), Q2 (*n* = 159; 84 boys, 75 girls), Q3 (*n* = 175; 84 boys, 91 girls), Q4 (*n* = 151; 78 boys, 73 girls) and Q5 (*n* = 172; 88 boys, 84 girls). School types with a Q1–Q3 status are considered to be schools located in low socio-economic (SES) sectors of the North West province.

### Measuring instruments

#### Anthropometric measurements

The anthropometric measurements included height (cm) and body mass (kg). These variables were measured by trained post-graduate students in Human Movement Sciences specialising in Kinderkinetics in accordance with standardised procedures. Two height measurements were taken by means of a portable stadiometer to the nearest 0.1 cm while standing barefoot and the mean of the two measurements was taken. Body mass was measured with an electronic scale (BF 511, Omron) to the nearest 0.1 kg. The body mass index (BMI) (kg/m^2^) was calculated using the height and body mass measurements.

**Determining *Z*-scores for stunting, wasting and underweight:** The WHO reference data (WHO 2007) were used as a standard to determine the HAZ, WHZ and *Z*-score BMI-for-age (BMIZ). *Z*-scores for height-for-age and weight-for-height of less than −2 standard deviations (s.d.) were used to determine stunting and wasting. The *Z*-score for underweight was determined using BMI-for-age, under the fifth percentile from the international reference population (De Onis et al. 2006). Larger negative *Z*-scores indicate higher levels of under-nutrition.

#### Bruininks–Oseretsky Test of Motor-Proficiency 2 Short Form

The Bruininks–Oseretsky Test of Motor-Proficiency 2 Short Form (BOT2-SF) was used to assess motor proficiency (MP). The BOTMP-SF consists of 14 items drawn from the eight sub-tests of the BOT-2 (Bruininks & Bruininks 2005) to span the broadest range of abilities and provide a single score of overall MP. The BOT2-SF evaluates motor skills in four area components which each consist of two sub-items: fine motor precision and fine motor integration, manual dexterity and bilateral coordination, balance and running speed and agility, upper limb coordination and strength. The scores are converted from point scores to standard scores. Administration of the assessment takes approximately 15–20 min and the BOT2-SF has proven to be a valid test to measure MP (Bruininks & Bruininks 2005).

#### Developmental Test of Visual Motor Integration fourth edition

The Beery–Buktenica Developmental Test of Visual Motor Integration (VMI 4th edition Beery 1997) was used to assess the visual motor skills of the participants. This test was developed to identify possible problems that children may have in specific areas of visual motor integration (VMI), the degree to which visual perception and finger–hand movements are well coordinated. *Two supplemental tests* focus on VP and *motor coordination* (MC), especially hand control in children between the ages of 3 and 18 years. The complete test takes approximately 10–15 min to administer. Children are required in the VMI test to copy a series of geometric figures, starting with simple figures and ending with complex figures. The copies are scored as successful (1) or failure (0) and scoring stopped when a child scored three consecutive failures. In the VMI-VP test, the task is to identify the exact match for as many as possible of the 27 geometric forms during a 3-min period. In the VMI-MC test which takes about 5 min to complete, the task is to simply trace the stimulus forms with a pencil without going outside double-lined paths. The three parts of the test were scored in sequence as required: firstly, the VMI, then the visual and then the motor part. The scores were totalled and converted to standard scores. The VMI test has been reported to be a culture-free and valid test (Beery 1997).

#### Teacher assessment: Academic performance

Basic academic literacy skills of Grade 1 subjects were assessed in 2010 by class teachers who used *The Mastery of Basic Learning Areas* assessment method. Numeric skills (mathematics) as well as reading and writing skills (standard of handwriting and pencil grip) were scored (CAPS Foundation 2011) according to a grading scale of one (1) to four (4), where (1) indicated no mastery, (2) partial mastery, (3) well mastered and (4) exceptional mastery.

### Procedure

It was indicated in the informed consent letter that participation was voluntary, with participants having the right to withdraw at any stage of the study. The research was conducted according to the guidelines of the Declaration of Helsinki.

#### Reliability and validity

All efforts were made to ensure reliable results. The study was considered to have good internal validity because of the rigorous study design and the care taken in the conducting of measurements. All researchers were well qualified to do the testing and one researcher was responsible for taking measurements throughout the gathering of the data process in order to yield consistent measurements. The external validity of the results was also considered appropriate because of the generalisability of the results. Test batteries that are used worldwide and also in SA were used to assess the motor functioning abilities of the subjects. Reliability of between *r* = 0.7 and *r* = 0.8 for different age groups are reported for the BOT-2 (Bruininks & Bruininks 2005), while the VMI-2 test has also been reported to be a culture-free and valid test (Beery 1997). All test administrators were post-graduate students and senior researchers in Kinderkinetics who were thoroughly trained theoretically and practically in conducting the measurements in these test batteries beforehand. All testers were also certified Level I Kinanthropometricts which verifies their capabilities to follow the anthropometric protocol of the International Society for the Advancement of Kinanthropometry (ISAK) (Stewart et al. 2011).

### Data analysis

The WHO Anthro Plus software (version 1.0.2, 2010) in SAS (Statistical Analysis System) was used to analyse the data. Data were firstly analysed for descriptive purposes by using means (m), standard deviation (s.d.), maximum and minimum values. The Pearson chi-square, *p* ≤ 0.05 and Cramer’s V were used to determine the statistical significance of overlap among stunting, wasting and underweight. Correlation coefficients were used to determine relationships among the different variables of interest where 0.1 indicates small practical significance, 0.3 moderate and 0.5 large practical significance. Independent *t*-testing was used to compare the groups with and without stunting, wasting and underweight. Differences were considered statistically significant at a *p* ≤ 0.05 level, and practical significance was determined by using *d*-values (Cohen 1988).

### Ethical considerations

Ethical approval for the execution of the study was obtained from the Ethics Committee of the North-West University (No. 00070 09 A1). Permission was also obtained from the Department of Basic Education and school headmasters and informed consent was obtained from the parents.

## Results

Table 1 shows the descriptive characteristics of the group regarding stunting, wasting and underweight. In the group, 4.29% (*n* = 35) were stunted, of which four showed severe stunting. Thirty-five (4.29%) were wasted, while 7.35% (*n* = 60) were underweight. These characteristics confirm moderate nutritional deficiencies in the group. The stunted group consisted of slightly more girls (17) than boys (18), while the wasted and underweight groups included more boys than girls. Cross-tabulation shows significant proportions of overlap between the HAZ and WHZ conditions (Pearson chi-square, *p* ≤ 01, Cramer’s *V* = 0.419) and also between WHZ and BMIZ (*p* ≥ 0.01, Cramer’s *V* = 0.297) which confirmed co-existence between these conditions. Proportions of overlap between WHZ and BMIZ were, however, not significant (*p* = 9397, Cramer’s *V* = 0.012).

**TABLE 1:** Characteristics of the group (*N* = 816) as categorised in stunted, wasted and underweight sub-groups.

Variable	Stunted				Wasted				Underweight			
	Severe		Stunted		Severe		Wasted		Severe		Underweight	
	*N*	%	*N*	%	*N*	%	*N*	%	*N*	%	*N*	%
Boys (*n* = 420)	3	0.72	14	3.34	1	0.24	24	5.73	1	0	34	4.77
Girls (*n* = 396)	1	0.25	17	4.28	0	0.00	10	2.52	0	0	25	1.00
All (*N* = 816)	4	0.49	31	3.80	1	-	34	4.19	1	-	59	2.94
Prevalence	-	-	35	4.29	-	-	35	4.29	-	-	60	7.35

*N*, number of participants; %, percentage.

Table 2 displays the correlation coefficients between the different HAZ, WHZ and BMIZ categories of under-nutrition and composite motor scores, VMI, VP and MC scores and the school performance of the group. Stunting and wasting correlated with all the outcome variables although stunting showed the highest correlations with all these variables. These correlations were mostly small (< 0.30) although a moderate practically significant relationship was found between VP and stunting (0.35) which was also the highest correlation that was established in the study. Except for one small practically significant relationship between underweight and visual perception (0.10), BMIZ did not correlate significantly with any of the outcome variables. Maths, reading and writing correlated with MP, VP, VMI and MC with the highest correlations that were found between VP and VMI (> 0.30) and school performance.

**TABLE 2:** Relationships between indices of under-nutrition and motor and academic performance.

Variables	HA*Z*	WH*Z*	BMI*Z*	Maths	Reading	Writing
BOT-short form SC	0.25*	0.19*	0.05	0.26*	0.25*	0.23*
Visual motor integration SC	0.26*	0.24*	0.09	0.38**	0.36**	0.38**
Visual perception SC	0.35**	0.29*	0.10*	0.37**	0.38**	0.33**
Motor coordination SC	0.23*	0.19*	0.09	0.25*	0.26*	0.26*
Maths	0.22*	0.19*	0.09	-	-	-
Reading	0.19*	0.16*	0.06	-	-	-
Writing	0.18*	0.16*	0.06	-	-	-

SC, standard score; HA*Z, Z*-score for height-for-age; WH*Z, Z*-score for weight-for-height; BMI*Z, Z*-score for body mass index-for-age.

* , *d* = 0.1, small practical significance;

** , *d* = 0.3, medium practical significance.

Tables 3–5 display significant differences between the mean standard scores obtained for school performance and MP, VMI and visual perceptual skills in the HAZ, WHZ and BMIZ categories compared to children without these conditions, referred to as typical children. The analysis of school performance differences (Table 3) revealed that mathematics performance of both HAZ (*t* = 2.01 *p* = 0.0446) and WHZ (*t* = 4.12, *p* = 0.000) groups were significantly poorer compared to typical children. No reading skills differences were found in the HAZ group, while writing skills differences reached borderline significance (*p* = 0.06) in this group. Of note is that the WHZ group displayed lower mean values for mathematics, reading and writing compared to HAZ children, and all three academic skills were also significantly poorer compared to those in non-wasted children (*p* < 0.05). The academic performance of underweight children did not differ from non-underweight children (*p* > 0.05) although their mean academic scores were slightly lower in all three measures. The total score for MP (BOT-SF, Table 4) indicated significantly poorer performances in the stunted (3.68, *p* < 0.01) and wasted groups (2.74, *p* = 0.0034), and also in most of the eight sub-items of MP. Of the eight sub-items, only balancing and running speed and agility of both the HAZ and WHZ groups, bilateral integration skills in HAZ children and fine motor precision and integration skills in WHZ children did not differ from typical children. Contrary to children presenting with stunting and wasting, no significant differences were found in the different MP outcome variables of underweight and normal weight children.

**TABLE 3:** Differences in visual motor integration, visual perception and motor coordination skills in children with and without nutritional deficits.

Variable	Stunted		Non-stunted		*t*	*p*-level	Wasted		Non-wasted		*t*	*p*-level	Underweight		Non-underweight		*t*	*p*-level
	Mean ±	s.d.	Mean ±.	s.d			Mean ±	s.d.	Mean ±	s.d.			Mean ±	s.d.	Mean ±	s.d.		
Maths	2.58	0.89	2.90	0.87	2.0100	0.0446*	2.29	0.94	2.92	0.86	4.1200	0.0000*	2.78	0.92	2.89	0.87	0.9600	0.3391
Reading	2.42	1.03	2.61	0.92	1.1500	0.2498	2.15	1.08	2.63	0.91	2.9900	0.0029*	2.52	0.85	2.61	0.93	0.7700	0.4422
Writing	2.52	0.96	2.81	0.88	1.8200	0.0689	2.26	0.99	2.82	0.87	3.6400	0.0003*	2.75	0.89	2.79	0.88	0.4200	0.6771

s.d., standard deviation; *t, t*-value; *p*-level.

* , *p* < 0.05 is significant.

**TABLE 4:** Differences in motor proficiency between children with and without nutritional deficits.

Variable	Stunted		Non-stunted		*t*	*p*-level	Wasted		Non-wasted		*t*	*p*-level	Underweight		Non-underweight		*t*	*p*-level
	Mean ±	s.d.	Mean ±	s.d.			Mean ±	s.d.	Mean ±	s.d.			Mean ±	s.d.	Mean ±	s.d.		
BOT-SF-SC	37.94	5.22	41.25	6.48	2.74	0.0034*	37.0	5.09	41.31	6.45	3.68	<0.001*	41.2	5.86	41.12	6.51	−0.10	0.9172
Fine motor precision	6.42	2.36	7.21	3.57	1.22	0.2225	6.0	2.10	7.21	3.57	1.97	0.0495*	7.92	3.05	7.12	7.12	−1.71	0.0875
Fine motor integration	1.68	1.42	2.05	1.91	1.06	0.2874	1.29	1.45	2.06	1.90	2.31	0.0213*	2.23	1.93	2.01	1.89	−0.89	0.3743
Manual dexterity	4.52	0.93	4.93	1.05	2.15	0.0321*	4.32	1.09	4.93	1.05	3.31	<0.001*	4.83	0.91	4.91	1.07	0.55	0.5813
Bilateral coordination	4.32	2.17	5.07	1.98	2.05	0.0404*	4.18	2.12	5.07	1.97	2.57	<0.001*	5.42	1.75	4.10	2.00	−1.57	0.1169
Balance	6.77	1.20	6.79	1.44	0.07	0.9441	6.79	1.45	6.79	1.43	−0.01	0.9910	6.58	1.65	6.82	1.42	1.16	0.2453
R-speed and agility	8.26	0.86	8.09	1.10	−0.83	0.4070	8.06	0.95	8.10	1.10	0.23	0.8196	8.12	1.04	8.10	1.10	−0.11	0.9122
Upper limb coordination	6.97	3.43	7.93	2.51	2.05	0.0404*	7.24	3.31	7.92	2.52	1.52	0.1278	7.78	2.39	7.89	2.58	0.31	0.7531
Strength	3.65	1.70	4.65	2.20	2.52	0.0120*	3.97	2.10	4.63	2.20	1.72	0.0865	4.68	1.85	4.60	2.22	−0.29	0.7683

BOT 2, Bruininks–Oseretsky test; SC, standard score; s.d., standard deviation; *t*, t-value; *p*-level.

* *p* < 0.05 is significant.

**TABLE 5:** Differences in visual motor integration, visual perception and motor coordination skills in children with and without nutritional deficits.

Variable	Stunted		Non-stunted		*t*	*p*-level	Wasted		Non-wasted		*t*	*p*-level	Underweight		Non-underweight		*t*	*p*-level
	Mean ±	s.d.	Mean ±.	s.d			Mean ±	s.d.	Mean ±	s.d.			Mean ±	s.d.	Mean ±	s.d.		
VMI SC	58.16	9.37	91.76	13.87	2.6200	0.0088*	87.18	12.44	91.66	13.80	1.8600	0.0631	94.02	13.42	91.27	13.78	1.4900	0.1378
VP-SC	67.81	20.21	79.86	23.05	2.8700	0.0042*	70.06	20.78	79.77	23.03	2.4200	0.0158*	80.98	22.98	79.24	23.01	0.5700	0.5716
MC-SC	87.32	14.05	93.23	14.68	2.2000	0.0281*	89.29	12.94	93.15	14.74	1.5000	0.1342	94.88	14.97	92.83	14.65	1.0400	0.2978

SC, standard score; s.d., standard deviation; VMI, visual motor integration; VP, visual perception; MC, motor coordination; *t, t*-value; *p*-level.

* , *p* < 0.05 is significant.

Visual motor integration skills, which were also assessed independently as visual perception (Table 4) and MC skills, were all significantly poorer in HAZ children compared to typical children, while only the VP skills of WHZ children were negatively influenced (*t* = 2.87 *p* = 0.0042). The mean scores of all these skills were also lower in HAZ children compared to WHZ children, especially VMI skills (58.16 vs. 87.18). No significant differences were found between the scores obtained by underweight and non-underweight children.

## Discussion

This study aimed to gain a better understanding of the association between different forms of under-nutrition on school performance of first-grade learners and how MP and sensory-related functioning skills such as VMI and VP of children with stunting, wasting and underweight relate to their academic performance. Two main findings emerged from the study. Definite relationships were firstly confirmed between stunting and wasting although mostly small in magnitude, and most of the school performance and other outcome variables, while we failed to establish links between underweight and any of these variables. This group was mostly characterised by mild forms of under-nutrition which might have contributed to the modest relationships that were found. Clear interrelationships were secondly also established between visual perception, VMI and MC skills and mathematics performance of stunted and wasted children, while only wasted children also experienced problems with reading and writing.

The impaired school performance and motor functioning of stunted and wasted children in this study agreed with other studies that confirmed such relationships (Glewwe et al. 2001; Milman et al. 2005; Sudfeld et al. 2015). Kar et al. (2008) reported an extensive range of deficits in cognitive functions and educational processes in stunted children, suggesting that the rate of development of cognitive functions may follow different patterns in stunted children. Our results, confirming correlations with school performance, ranging between 0.18 and 0.21, with the highest correlation with mathematics (0.24), also concur with a pooled summary correlation score of 0.28 which is based on the findings of 11 studies regarding the HAZ and cognition relationship (Sudfeld et al. 2015). Studies of stunted children in LMIC in Africa also confirm positive relationships with mathematical performance (Themane et al. 2003) where Ghandi et al. (2011) found that height gain in Malawian children between 18 and 60 months predicted maths performance at 12 years.

Although the correlations with academic skills were of a smaller magnitude in wasted children, their reading and writing literacy skills differed significantly from non-wasted children, which was not the case in stunted children. The mean scores that wasted children obtained for maths, reading and writing were also poorer in all these three school performance areas compared to in stunted children. No studies could be found that compared similar variables that we studied in wasted children. We did, however, establish a significant proportion of overlap between the children who were categorised in the stunting and wasting groups and wasted children also had significantly poorer strength and upper limb coordination than typical children, which was not the case in the stunted group. These additional MC and physical fitness handicaps could have contributed to the added academic risk in the wasted children as seen in fine motor skills (Van der Fels et al. 2015). Wasting can be a chronic but also an acute form of malnourishment or the result of poor health. Researchers reported in this regard that iron is needed for the development but also the functioning of the human brain (Nyaradi et al. 2013). A chronic form of a micronutrient deficiency such as iron anaemia can subsequently contribute to poor concentration in wasted children and can also affect motor development (Black et al. 2013).

It is also reported that malnourished children usually have less energy and interest in learning and experience more behavioural problems such as negative effect, apathy and reduced activity, while they are predisposed to a lower IQ (Liu et al. 2004) which again predisposes them to externalising behaviour problems such as hyperactivity behaviour. All these factors can thus negatively influence learning effort and school attendance (Glewwe et al. 2001), and also influence children’s reading and maths and fine motor precision in performing writing skills.

Spernak et al. (2006) report that the largest effect of poor health longitudinally was on the reading skills of Grade 3 learners. It might thus be that children in this study who were not only stunted, but also wasted and who might hence also suffer from acute illnesses, might have an added risk for academic difficulties.

Our results further suggest an association between the motor skills deficiencies and visual perception problems and cognitive impairments of stunted and wasted children that were mostly associated with maths difficulties. There is a growing body of evidence that confirms a mutual association between cognition and motor skills (Abe & Hanakawa 2009; Hanakawa 2011). Neuro-imaging techniques have shown that regions important to motor performance and cognition such as the cerebellum, dorso-lateral prefrontal cortex and the connecting structures including the basal ganglia are co-activated in certain motor and cognitive tasks, thus confirming the mutual association between these two domains (Abe & Hanakawa 2009; Hanakawa 2011). Furthermore, it is also reported that areas of the brain implicated in language functions such as the Broca’s area are activated during motor tasks. Van der Fels et al. (2015), however, reported that although motor, cognitive and language development may share several underlying processes, specifically fine motor skills showed the strongest relationship with higher order cognitive skills in their study. The embodied cognition theory (Thelen 2000) proposes that motor skills constrain the development of other skills. This theory proposes that all cognition including high-level processes like the language process relies on internal stimulation of the sensory motor experience which confirms a close link between motor skills and cognitive information processing and subsequently an interplay between language, motor acts and the motor system. A meta-analysis published by Sudfeld et al. (2015) also reported a pooled summary correlation score of 0.24 obtained from four cross-sectional studies between motor scores and HAZ in children from LMIC which agrees with the correlations that we obtained between HAZ and WAZ and the different motor scores (ranging between 0.22 and 0.34). A growing body of evidence (Davis et al. 2011) suggests that motor skills development impacts on many other areas of academic performance and especially early school success. Davis et al. (2011) also report that children with poor motor skills generally have co-existing difficulties which can increase the risk of academic difficulties. It is thus assumed that if a young child’s motor skills are atomised, they will need less energy to concentrate on performing these skills and can instead concentrate on the academic tasks at hand.

Visual motor integration and VP of stunted children were significantly inferior to typical children. Visual perception also correlated the highest with stunting (0.34) and with wasting (0.29), and were significantly impaired in HAZ and WAZ children. Studies relate VMI to cognitive performance and influences on intelligence coefficients (Duijff et al. 2012), general academic performance and maths and writing skills (Son & Meisels 2006) are reported. Decker et al. (2011) hold the view that the developmental trend that links cognitive abilities to quantitative and non-verbal reasoning abilities shows a correlation with visual motor skills among four- to seven-year-olds. Poorer memory and visual processing skills are reported, while similar relationships with cognitive performance have also been confirmed for VP by Grantham-McGregor et al. (1997). Laus et al. (2011) indicated that even mild but persistent malnutrition during early life can contribute to impaired visio-spatial functions which are known to have an influence on the understanding of mathematical concepts. Bezrukikh and Terebova (2009) are of the opinion that visual perception forms the basis of a child’s cognitive activity, because it orientates and controls a child’s behaviour. Visual perception involves various structures of the brain, each of which makes a specific contribution to active perception (Bezrukikh & Terebova 2009). The cerebellum and the parietal cortex are involved in spatial and numerical perceptions which are both important for mathematics tasks (Bueti & Walsh 2009). Farber and Beteleva (2005) report a rapid development of the brain’s cortical areas and intra-cortical connections between 5 and 7 years, which is considered an important developmental age period for visual perception skills. The cerebral structures involved in visual perception, however, do not reach maturity simultaneously during this developmental period with different parts and functions of the brain that develop at different times (Grossman et al. 2000). Under-nutrition is reported to affect areas of brain development involved in cognition, memory and locomotor skills. Brown and Pollit (1996) report that cognitive and perception functions make use of similar brain structures such as the cerebellum and the basal ganglia. The underperformance in both VP and academic tasks, especially mathematics, in our group could thus suggest possible structural damage to the brain in these areas. Stunting and wasting are linked to damage to the brain of which the effect may vary according to the severity, duration and the timing of nutritional deprivation and which can be irreversible in severe cases (Benefice et al. 1996). Micronutrients such as iron and iodine are needed for the development and functioning of the human brain and deficiencies in this regard can affect the structure and functioning of the brain (Nyaradi et al. 2013). This can also suggest improper functioning of the central information processing mechanisms of the brain (Sherrill 2004) which can contribute to poor sensory recognition, awareness or perception which is said to affect any part of the body or a particular sense, for example body, space, time, object, movement and touch which are incorrectly perceived, thus contributing to perceptual disorders that influence cognition. Kumar (2007) reported that iodine and anaemia deficiencies can affect cognitive performance, behaviour and motor development, coordination, language development and scholastic achievements. The co-existence of poor visual perceptual functioning and maths problems in this group could thus be a result of damage to similar structural areas of the brain that are involved in cognition and VP and this may contribute to a compounded risk of having neuro-developmental problems (Prendergast & Humphrey 2014) in stunted and wasted children.

No significant differences were found between the motor functioning of the underweight and normal weight children. This might be because underweight often detects acute changes associated with wasting, rather than a chronic condition which has fewer long-term consequences. Black et al. (2013) reported in this regard different levels of causation for optimum growth and development, where food, disease and optimal care play a crucial role at the proximal level. Weight-for-age is primarily a composite index of both weight-for-height and height-for-age and fails to distinguish tall, thin children from those who are short with adequate weight. In support of our findings, the use of weight-for-age in predicting or identifying ‘wasted’ children was found to have a low sensitivity and specificity in three US populations. Black et al. (2013) reported that iron deficiency anaemia affects motor development and has effects on cognitive development and this was more clearly shown in children older than 5 years than in younger children. However, a tendency towards lower mean values for literacy in maths, reading and writing were found in the underweight group.

## Conclusions, limitations and recommendations for future research

This study confirmed an inverse relationship between moderate indices of stunting and wasting and school performance in maths, reading and writing of school beginners, highlighting barriers to academic achievement which should be alleviated. In addition, poorer MP and visual-perception functioning were also found in both HAZ and WHZ conditions. Children require the coordination of many different skills to be successful in school. These early motor performance skills, and especially visual perception functioning that correlated with HAZ, WAZ and BMIZ, are recognised as important contributing factors to academic readiness of young school beginners. As academic achievement is important for future personal health and well-being, it is therefore of significant concern for public health. The high prevalence of under-nutrition worldwide but especially in low- to middle-income countries (LMIC), such as SA, where this study was conducted, necessitates the need to institute programmes to improve school age children’s cognitive development through various mechanisms. It is, however, important to remember that under-nutrition most probably occurs with other risks such as health conditions that are associated with under-nutrition and inadequate stimulation at home by parents, but also by caregivers in caring facilities, to mention a few. Identifying the modifiable factors is critical to planning therapeutic interventions and a comprehensive understanding of the child’s abilities and limitations. Under-nutrition should therefore receive attention using a multi-sectorial approach where stakeholders from different disciplines should be identified to improve the condition and the adverse effects on children’s overall development and especially scholastic performance. Routine assessment of both weight and height in all children needs to become standard clinical practice from very early childhood onwards as the early recognition of insufficient weight gain relative to linear growth is essential, while learning opportunities, motor stimulation and improved awareness among parents and caregivers about the negative consequences of this condition, in addition, good micronutrient nutrition, are also needed to improve early childhood development. There is a need for longitudinal research, especially regarding the pathways of how nutritional status influences learning in especially developing countries, in order to gain a better understanding of how to assist these children to attain their full developmental potential.

This study had limitations that should be acknowledged. It was based on cross-sectional and regional data; therefore, cause and effect could not be established and the results therefore have shortcomings. The influence of different teachers in the school performance results could also not have been ruled out in the results. Although it is reported that stunting and wasting mostly co-exist, the strong points of this study are that the different forms of under-nutrition, which also includes underweight, were assessed separately in this study and therefore add to the understanding of the differential influences of these conditions on early childhood development and school performance. The strength of the study is, however, that the results are based on a big (*N* = 816) and random sample of children who make the findings more generalisable.
